# Gradient nonlinearity correction in liver DWI using motion-compensated diffusion encoding waveforms

**DOI:** 10.1007/s10334-021-00981-6

**Published:** 2021-12-11

**Authors:** Sean McTavish, Anh T. Van, Johannes M. Peeters, Kilian Weiss, Marcus R. Makowski, Rickmer F. Braren, Dimitrios C. Karampinos

**Affiliations:** 1 Department of Diagnostic and Interventional Radiology, School of Medicine, Klinikum rechts der Isar, Technical University of Munich, Munich, Germany; 2 Philips Healthcare, Best, The Netherlands; 3 Philips Healthcare, Hamburg, Germany

**Keywords:** Diffusion-weighted imaging, Gradient nonlinearity, Motion compensation, ADC mapping, Liver imaging

## Abstract

**Objective:**

To experimentally characterize the effectiveness of a gradient nonlinearity correction method in removing ADC bias for different motion-compensated diffusion encoding waveforms.

**Methods:**

The diffusion encoding waveforms used were the standard monopolar Stejskal–Tanner pulsed gradient spin echo (pgse) waveform, the symmetric bipolar velocity-compensated waveform (sym-vc), the asymmetric bipolar velocity-compensated waveform (asym-vc) and the asymmetric bipolar partial velocity-compensated waveform (asym-pvc). The effectiveness of the gradient nonlinearity correction method using the spherical harmonic expansion of the gradient coil field was tested with the aforementioned waveforms in a phantom and in four healthy subjects.

**Results:**

The gradient nonlinearity correction method reduced the ADC bias in the phantom experiments for all used waveforms. The range of the ADC values over a distance of ± 67.2 mm from isocenter reduced from 1.29 × 10^–4^ to 0.32 × 10^–4^ mm^2^/s for pgse, 1.04 × 10^–4^ to 0.22 × 10^–4^ mm^2^/s for sym-vc, 1.22 × 10^–4^ to 0.24 × 10^–4^ mm^2^/s for asym-vc and 1.07 × 10^–4^ to 0.11 × 10^–4^ mm^2^/s for asym-pvc. The in vivo results showed that ADC overestimation due to motion or bright vessels can be increased even further by the gradient nonlinearity correction.

**Conclusion:**

The investigated gradient nonlinearity correction method can be used effectively with various motion-compensated diffusion encoding waveforms. In coronal liver DWI, ADC errors caused by motion and residual vessel signal can be increased even further by the gradient nonlinearity correction.

**Supplementary Information:**

The online version contains supplementary material available at 10.1007/s10334-021-00981-6.

## Introduction

Diffusion-weighted imaging (DWI) remains a valuable tool in liver lesion detection and there is an ongoing interest in ADC mapping for tumor staging and therapy monitoring [1, 2]. However, cardiac and respiratory motion from the subject during the standard monopolar Stejskal–Tanner-pulsed gradient spin echo (pgse) diffusion encoding gradients can cause intravoxel dephasing and therefore signal loss [3–8]. Since the apparent diffusion coefficient (ADC) is proportional to the diffusion-induced signal decay when diffusion encoding is applied, any undesired motion-induced signal loss will, therefore, cause an overestimation of the ADC. The close proximity of the left liver lobe to the heart makes the left liver lobe especially susceptible to cardiac motion-induced signal loss [7].

Respiratory-triggered scans are most frequently used to overcome issues caused by the breathing motion of the subject in liver DWI [3, 4, 9–12]. Cardiac-triggered scans have also been proposed to overcome the issues caused by cardiac motion. However, cardiac-triggered DWI scans are associated with low acquisition efficiency and require the optimisation of the trigger delay at each individual subject [13]. In addition, it has been recently suggested that a single trigger delay might not be adequate for removing artifacts induced by cardiac motion and vessel pulsation throughout the whole liver [14, 15]. Therefore, motion-compensated diffusion gradient encoding waveforms have recently been proposed as alternatives for motion-robust liver DWI [15–24].

**Fig. 1:**
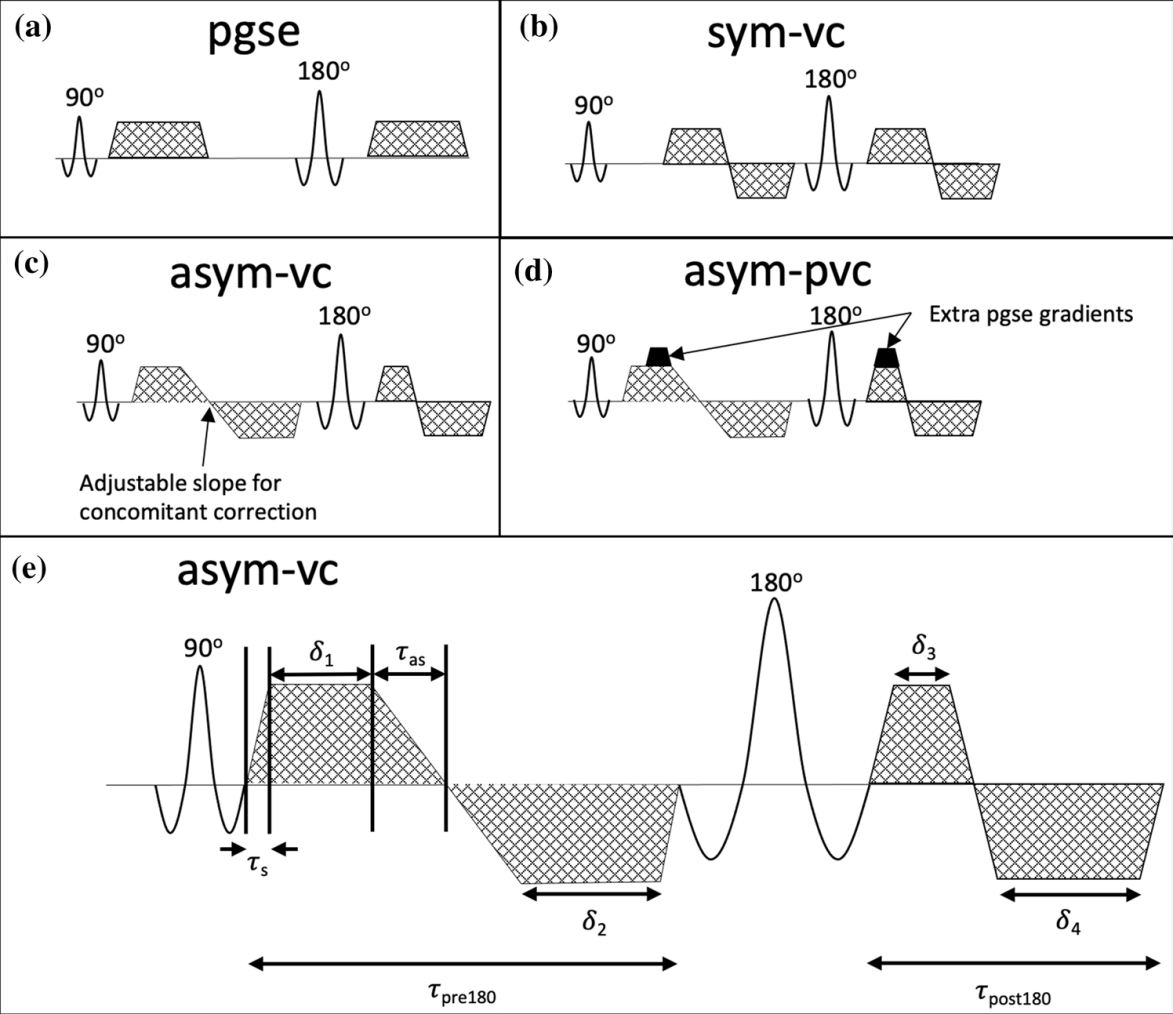
Diffusion encoding waveform designs for pgse, sym-vc, asym-vc and asym-pvc. sym-vc is symmetric and therefore does not have an optimal echo time. asym-vc is optimized for echo time by making the lobes asymmetrical. asym-pvc takes the m1-nulled design of asym-vc and adds small pgse gradients (at *b*-values other than the maximum *b*-value) to increase the *m*_1_ value. At the highest *b*-value, the increased m_1_ is achieved by adjusting the relative lengths of each lobe

The simplest motion-compensated diffusion encoding waveform that has been proposed is a symmetric bipolar waveform (sym-vc). An example of the sym-vc waveform design is given in Fig. 1b. In this waveform, the gradient first moment (${m}_{1}=\gamma \int tG\left(t\right)dt$) is zero, leading to no phase accumulation and hence no signal loss when spins move with constant velocities during diffusion encoding. When an echo planar imaging (EPI) readout is used in combination with the symmetric bipolar waveform, the unavoidable deadtime between the excitation and refocusing pulses means that this waveform is not optimal in terms of echo time. Various other diffusion encoding waveforms that use an asymmetric design to optimize the echo time have recently been proposed [15–20]. The ODGD [19] and CODE [16] formulations use constrained optimization algorithms to find arbitrarily shaped echo time optimized diffusion encoding waveforms.

When using asymmetric diffusion encoding waveforms (an example of one such waveform is given in Fig. 1c), spatially dependant concomitant gradient fields, which are characterized by Maxwell’s equations, can induce dephasing and therefore signal loss over the field of view in DWI sequences [25, 26]. These concomitant gradients must therefore be accounted for either in the pulse sequence design or with postprocessing. One issue with both the symmetric and asymmetric motion-compensated *m*_1_-nulled waveforms is that the vessel suppression capability is decreased and *T*_2_ shine through is increased [15, 20]. Therefore, vessels in liver DWI acquired with motion-compensated waveforms can appear bright, which can confound lesion detection and cause errors in ADC quantification. To address the bright vessel signal problem, adding *m*_1_ > 0 back to the motion-compensated waveforms (sym-vc or asym-vc) has been proposed to find a balance between motion sensitivity and vessel signal suppression [15, 20, 27, 28]. An example of one such asymmetric partial velocity-compensated waveform is given in Fig. 1d.

Another source of acquisition-related errors in ADC estimation is gradient nonlinearity, which is a static effect specific to the gradient coil design. With imaging gradients, gradient nonlinearity deviates the relationship between the spatial position and acquired data frequencies, leading to spatial image warping [29, 30]. With diffusion gradients, gradient nonlinearity causes the *b*-value to be different than intended and can cause ADC quantification errors larger than 10% in clinically relevant field of views [31]. Specifically, since the calculation of the *b*-value in DWI relies on a spatially uniform diffusion-weighted gradient, any spatially non-uniform gradient leads to errors in the calculation of the *b*-value and, therefore, to ADC bias [31].

Previous works have proposed methods to correct for gradient nonlinearity effects in diffusion MRI [31–34]. Bammer et al. formulated a general mathematical framework characterizing errors from gradient nonlinearity [32, 33]. The gradient field was approximated with a spherical harmonic expansion, which was then used to derive a gradient nonlinearity tensor, enabling the correction of both the magnitude and direction of the gradients. Although comprehensive, the above approach requires the acquisition of at least six diffusion directions. This is commonplace in diffusion tensor imaging (DTI) experiments, but in many body diffusion applications DWI protocols based on three orthogonal directions are typically acquired. Malyarenko et al. have proposed a simplified scheme to correct ADC bias due to gradient nonlinearity in DWI protocols employing three orthogonal directions [33]. The scope of the correction proposed by Malyarenko et al. was limited to DWI applications and was, therefore, not suitable for DTI applications [33]. The correction involved the rotation of the gradient nonlinearity tensor into the diffusion gradient frame, where the spatial bias of the *b*-matrix was approximated by the Euclidean norm. Although it has been theoretically predicted to work for arbitrary waveform designs, none of the previous works to correct ADC bias due to gradient nonlinearity in body DWI have been experimentally applied to any diffusion encoding gradient waveforms other than the conventional monopolar Stejskal–Tanner pgse diffusion encoding gradient waveform. Therefore, it is yet to be experimentally verified that the gradient nonlinearity correction method based on the work by Malyarenko et al. will be equally as effective for motion-compensated diffusion encoding waveforms as for the conventional monopolar diffusion encoding waveform.

The aim of the present work is to assess the effectiveness of the gradient nonlinearity correction for different motion-compensated diffusion encoding waveforms and specifically to characterize the effectiveness of the gradient nonlinearity correction in removing ADC bias due to gradient nonlinearity for different motion-compensated diffusion encoding waveforms in the presence of motion effects in the context of ADC mapping in the liver.

## Methods

### Motion-compensated diffusion encoding waveforms

The following diffusion encoding waveforms were presently investigated (Fig. 1).

#### Pulsed gradient spin echo waveform (pgse)

The standard monopolar Stejskal–Tanner pgse diffusion encoding waveform (Fig. 1a) was first used as it remains the most frequently used diffusion encoding waveform in body DWI.

#### Symmetric velocity-compensated waveform (sym-vc)

In the symmetric bipolar waveform (sym-vc), shown in Fig. 1b, the gradient first moment (${m}_{1}=\gamma \int tG\left(t\right)dt$) is zero.

#### Asymmetric velocity-compensated waveform (asym-vc)

When an echo planar imaging (EPI) readout is used in combination with the symmetric bipolar waveform, the unavoidable deadtime between the excitation and refocusing pulses means that this waveform is not optimal in terms of echo time. An asymmetric velocity-compensated diffusion encoding waveform with optimized echo time was then used. The adoption of asymmetric diffusion encoding waveforms to minimize the echo time requires the additional consideration of concomitant gradient effects, which do not cancel out as in the case of symmetric diffusion encoding waveforms [25, 35, 36]. Different strategies have been previously performed to define velocity-compensated diffusion encoding with optimal TE and correction of concomitant field effects, including strategies that approximate the concomitant phase as a linear phase variation [16] and strategies that null the concomitant gradient-induced phase accrual [19]. Specifically, by optimizing the *b*-value formulation for arbitrary gradient waveforms with appropriate constraints on system hardware, sequence timings, concomitant fields, and gradient moments, the optimized diffusion-weighting gradient waveform design (ODGD) defined a TE-optimized concomitant field corrected motion-compensated waveform [19]. However, since the optimization for the ODGD waveform must typically be done offline (not at the scanner), a file that contains the optimized waveform has to be loaded onto the scanner, reducing in general the flexibility for protocol optimization.

The presently employed asymmetric velocity-compensated (asym-vc) waveform builds upon the ODGD formulation [19]. Without concomitant field correction, the optimal ODGD waveform is always a collection of trapezoids. Additionally, for a wide range of relevant sequence parameters in the employed *b*-value range, the number of trapezoid lobes and polarities of an optimized waveform do not change. Therefore, the optimization of the waveform can be reduced to the optimization of the trapezoid lengths, which can be integrated into the pulse sequence environment and carried out efficiently online at the scanner. When concomitant field correction is added, the ODGD formulation yields optimal waveforms that are not trapezoidal. Alternatively, by making the slope of the trapezoidal waveform a variable and adding the concomitant field correction as a constraint, first-order concomitant field correction is applied by adjusting the slopes of the trapezoids until the concomitant field term is nulled. This second concomitant correction approach is similar to Zhang et al. [15] and Szczepankiewicz et al. [23, 24] and is suitable for the waveform optimization at the scanner.

The design of the asym-vc waveform is as follows. The asym-vc waveform is assumed to have four lobes, two before and two after the refocusing pulse. Figure 1e shows a labelled diagram of the asym-vc waveform, where ${\delta }_{1}, {\delta }_{2}, {\delta }_{3}$ and ${\delta }_{4}$ are the durations of the plateaus of each lobe, ${\tau }_{\mathrm{s}}$ is the duration of the non-adjustable slope, ${\tau }_{\mathrm{as}}$ is the duration of the adjustable slope and ${\tau }_{\mathrm{pre}180}$ and ${\tau }_{\mathrm{post}180}$ are the durations available before and after the refocusing pulse, respectively. The non-adjustable slopes are constant and are equal to the maximum slew rate. The other constraints for the timing of each lobe are the *m*_0_, *m*_1_ and concomitant constraints. The variables to solve for, in terms of the amount of time before and after the refocusing pulse, are the duration of each plateau of each lobe and the slope in between the first and second lobes. The constraints are therefore

1 $${\delta }_{1} +{\delta }_{2}+2{\tau }_{\mathrm{s}}+ {2\tau }_{\mathrm{as}}= {\tau }_{\mathrm{pre}180},$$

2 $${\delta }_{3} +{\delta }_{4}+4{\tau }_{\mathrm{s}}= {\tau }_{\mathrm{post}180},$$

3 $${m}_{0}=\gamma \int_{\tau }G\left(t\right)dt=0,$$

4 $${m}_{1}=\gamma \int_{\tau }tG\left(t\right)dt=0,$$

5 $$\gamma \int_{A1}{G(t)}^{2}dt-\gamma \int_{A2}{G\left(t\right)}^{2}dt=0,$$

where the integrals of ${m}_{0}$ and ${m}_{1}$ are over the entire duration of the gradient waveforms, given by $\tau$, and $A1$ and $A2$ are the time periods before and after the refocusing pulse, respectively. Equation 5 is the constraint imposed to minimize concomitant fields. With these five constraints, the five variables ${\delta }_{1}, {\delta }_{2}, {\delta }_{3}, {\delta }_{4}$ and ${\tau }_{\mathrm{as}}$ can be found.

To obtain the asym-vc waveform, the *b*-value equation is expressed in terms of the gradient lobe timings, which is then input into the scanner software environment and the echo time is optimized for a given *b*-value. ${\tau }_{\mathrm{pre}180}$ and ${\tau }_{\mathrm{post}180}$ are found from each so-called “target echo time” in the optimization loop. As the asym-vc waveform is restricted to trapezoidal waveforms and not arbitrarily shaped waveforms, the optimization procedure is not burdened by long computational times unsuitable for the direct scanner interface. Therefore, asym-vc waveform can be designed on-the-fly on the scanner, instead of having to input a text file containing the offline optimized waveform whenever scan parameters are changed. The resulting waveform has an asymmetric bipolar design as shown in Fig. 1c.

A further description about the differences between the implementation of the asym-vc, and representatives of existing optimized velocity-compensated waveforms such as the ODGD and the CODE [16] waveforms are included in the Supplementary Material.

#### Asymmetric partially velocity-compensated waveform (asym-pvc)

Compared to the pulse field gradient encoding (pgse) waveform, the sym-vc and asym-vc waveforms, while decreasing sensitivity to motion, also decrease the vessel suppression capability and increase *T*_2_ shine through [15, 20]. Therefore, vessels in liver DWI acquired with sym-vc and asym-vc can appear bright, which can confound lesion detection and cause errors in ADC quantification. The fact that the vessels can appear bright is caused in part by nulling the gradient first moment, *m*_1_. By adding a small *m*_1_ > 0 back to the motion-compensated waveforms (sym-vc or asym-vc) significant reduction of vessel signals can be achieved [15, 20, 27, 28]. The key idea of these methods is to find an appropriate *m*_1_ > 0 value that balances sensitivity to motion and vessel signal suppression. In the present work, small pgse gradients corresponding to the desired value of m_1_ were added to two of the gradient lobes of the asym-vc waveform for all *b*-values except for the maximum *b*-value, similar to Zhang et al. [15]. At the maximum *b*-value, in the present implementation, the lengths of each of the gradient lobes and the amplitude of the asym-vc gradients were adjusted until the desired value of *m*_1_ was obtained without changing the *b*-value. Although not fully TE-optimized, the above asymmetric partial motion-compensated waveform (asym-pvc) can be again designed on-the-fly at the scanner, facilitating protocol optimization. The asym-pvc waveform is shown in Fig. 1d. A detailed analysis of the employed asymmetric partially velocity-compensated waveform design is provided in the supplementary material, with a diagram shown in Fig. S1.

### Gradient nonlinearity correction

**Fig. 2:**
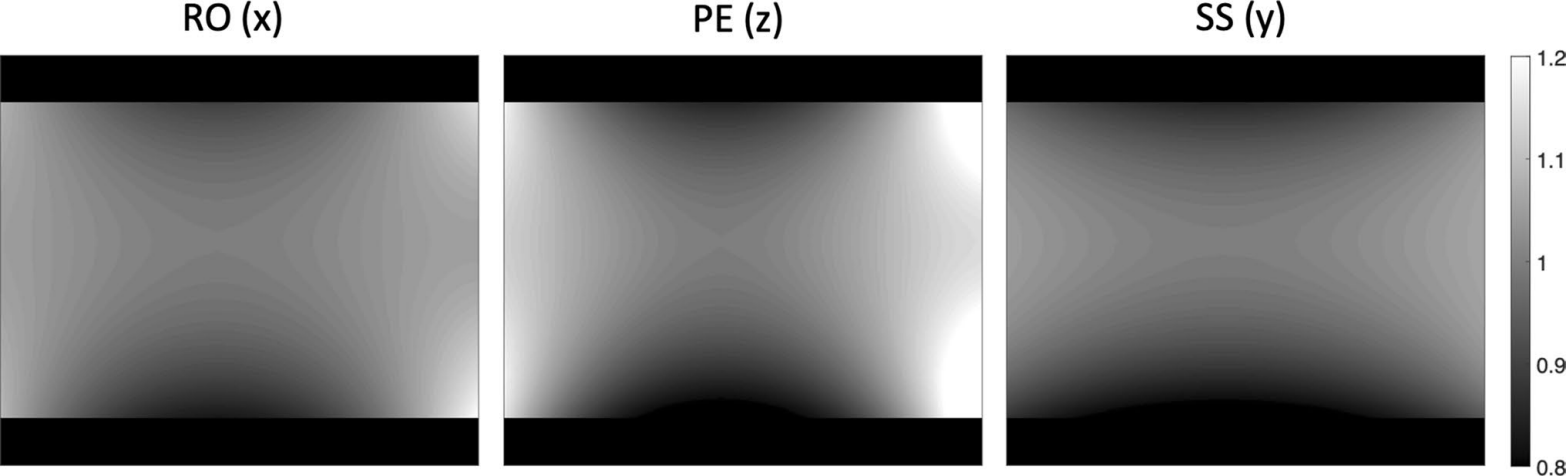
*b*-value correction maps for diffusion directions RO, PE and SS from one coronal slice of one of the in vivo measurements. The scale shows the ratio between the corrected and uncorrected *b*-value. The same correction maps were used for all waveforms. The scanner axes are given in brackets

The gradient nonlinearity correction (gnl) framework as proposed by Malyarenko et al. was adopted [33]. The method is briefly described here: in the case of linear gradients, the gradient strength is uniform across the whole magnet bore and does not depend on position. In the case of nonlinear gradients, the gradient strength is dependent on position within the magnet bore and spurious gradients orthogonal to the applied gradient will be produced. First-order spatial variations of the gradient can be described by a nonlinearity tensor ${\varvec{L}}({\varvec{r}})$, where

6 $${\varvec{g}}\left({\varvec{r}}\right)= {\varvec{L}}\left({\varvec{r}}\right){{\varvec{g}}}_{0}, \mathrm{where}\space\boldsymbol{ }\boldsymbol{ }{l}_{ij}\left({\varvec{r}}\right)= \frac{1}{\parallel {g}_{i0}\parallel }\frac{\partial {B}_{z}^{{g}_{i}}}{\partial {r}_{j}} , \boldsymbol{ }\left({\varvec{L}} \ne {{\varvec{L}}}^{T}\right),$$

where ${{\varvec{g}}}_{0}$ is the gradient at isocenter, ${\varvec{r}}$ is the spatial position from isocenter and ${\varvec{g}}\left({\varvec{r}}\right)$ is the spatially varying gradient. $\partial {B}_{z}^{{g}_{i}}$ is the *z* component of the magnetic field as a result of applying the gradient in the *i* direction and ${g}_{i0}$ is the *i* component of the gradient ${{\varvec{g}}}_{0}$. The spatially dependent magnetic field produced by each gradient coil can be described by a spherical harmonic expansion. Using the spherical harmonic coefficients provided by the manufacturer, the spatially varying magnetic field produced by each coil and, therefore, the gradient nonlinearity tensor ${\varvec{L}}$ can be derived. The spatially varying b matrix is given by ${{\varvec{b}}}^{^{\prime}}\left({\varvec{r}}\right)={\varvec{L}}\left({\varvec{r}}\right){{\varvec{b}}}_{0}{{\varvec{L}}}^{{\varvec{T}}}\left({\varvec{r}}\right)$, where ${{\varvec{b}}}_{0}$ is the nominal *b* matrix and includes *b* components due to the diffusion gradients, imaging gradients and imaging cross terms. The correction map is then obtained from (with further details given in [33])

7 $$C^{k} \left( {\varvec{r}} \right) = \frac{{\left\| {\varvec{b^{\prime}}^{k} ({\varvec{r}})} \right\|_{F} }}{{\left\| {{\varvec{b}}_{0}^{k} } \right\|_{F} }} \cong \frac{{\sqrt {Tr\left\{ {\left[ {\varvec{b^{\prime}}^{k} \left( {\varvec{r}} \right)} \right]^{2} } \right\}} }}{{b_{0}^{k} }} \cong \left[ {{\varvec{u}}_{k}^{T} {\varvec{L}}\left( {\varvec{r}} \right){\varvec{u}}_{k}^{ } } \right]\left[ {{\varvec{u}}_{k}^{T} {\varvec{L}}^{T} \left( {\varvec{r}} \right){\varvec{u}}_{k}^{ } } \right],$$

for each diffusion direction *k*, where $Tr\left\{.\right\}$ is the trace operator, $F$ is the Frobenius norm and ${{\varvec{u}}}_{k}$ is a unit vector that defines the *k*th DW direction in the gradient coil coordinates. The corrected *b*-value map is given by

8 $${b}_{c}^{k}\left({\varvec{r}}\right)= {b}_{n}^{k}{C}^{k}\left({\varvec{r}}\right),$$

where ${b}_{c}^{k}\left({\varvec{r}}\right)$ is the corrected spatially dependent *b*-value map for each diffusion direction k, ${b}_{n}^{k}$ is the nominal *b*-value and ${C}^{k}({\varvec{r}})$ is the correction map. Example correction maps are given in Fig. 2.

The code used to calculate the correction maps was provided by the vendor and was integrated into the reconstruction platform on the scanner. The individual *b*-values and directions as well as the design parameters of the gradient coil and slice orientations were input to the reconstruction process, after which the correction maps were generated as a reconstruction output of the diffusion scans. The ADC maps were computed in MATLAB from the correction maps and the diffusion-weighted data exported from the scanner.

### MRI measurements

All MRI measurements were performed on a 3 T Ingenia Elition X scanner (Philips Healthcare, Best, Netherlands), which has a maximum gradient amplitude of 45 mT/m and a maximum slew rate of 200 T/m/s.

#### Phantom measurements

Phantom validation measurements were performed to assess the performance of the gradient nonlinearity correction with the pgse, sym-vc, asym-vc and asym-pvc waveforms. A cylindrical phantom of length 200 mm and diameter 115 mm containing 31.5 mmol/L of NiCl_2_-6H_2_O was placed inside an extremity coil at isocenter and axial slices were acquired with a ss-EPI sequence in the S/I direction to investigate the gradient nonlinearity in the S/I direction. A separate scan in which sagittal slices in the L/R direction were acquired to investigate the gradient nonlinearity in the L/R direction was performed. More information is given in the supplementary material. Gradient nonlinearity was not measured in the A/P direction due to the unsuitability of the dimensions of the phantom for measuring in this direction.

For all waveforms in the axial slice experiment, seventeen slices were acquired with a FoV of 125 × 125 mm^2^, a voxel size of 2 × 2 × 6 mm^3^ and a 2.4 mm slice gap. The parallel imaging acceleration factor *R* = 2, effective bandwidth over phase encode direction = 57 Hz/pixel. *B*-values of [0, 600] s/mm^2^ were used for the ADC calculation, with eight averages per *b*-value and three orthogonal diffusion encoding directions along the readout (RO), phase encoding (PE) and slice selection (SS) directions. The echo times were 60/90/90/96 ms for pgse, asym-vc, asym-pvc and sym-vc, respectively. TR = 8000 ms in all cases and no partial Fourier encoding was used for any of the waveforms. An m_1_ value of 0.1 s/mm was used for the asym-pvc waveform.

ADC maps were calculated for each individual diffusion direction, as well as for all directions combined. An ROI was drawn in the center of each slice and the mean ADC over the ROI was calculated.

A separate scan was performed to test the performance of the concomitant gradient correction. The same phantom was placed inside the extremity coil and seventeen axial slices were acquired with a FoV of 128 × 128 mm^2^, a voxel size of 2 × 2 × 6 mm^3^ and a 2.5 mm slice gap. This scan was performed without parallel imaging and also with a parallel imaging factor of *R* = 2. When a parallel imaging factor of *R* = 2 is used, the number of acquired *k*-space lines is reduced in comparison to when no parallel imaging is used. Therefore, the time interval available for diffusion encoding after the refocusing pulse is closer to the time interval before the refocusing pulse, meaning that the diffusion encoding waveforms before and after the refocusing pulse are more symmetrical. Since concomitant gradient effects are affected by the asymmetry of the waveforms, using a parallel imaging factor of *R* = 2 was expected to reduce the effect of the concomitant gradients. For both scans, TR = 5000 ms, *b*-values of [0, 600] s/mm^2^ were used for the ADC calculation, with five averages per *b*-value and three orthogonal diffusion encoding directions along the readout (RO), phase encoding (PE) and slice selection (SS) directions. For the scan without parallel imaging, effective bandwidth over phase encode direction = 27.2 Hz/pixel, the echo times were 98/103 ms for asym-vc with and without concomitant gradient correction, respectively. For the scan with a parallel imaging factor of *R* = 2, effective bandwidth over phase encode direction = 54.5 Hz/pixel, the echo times were 89/90 ms for asym-vc with and without concomitant gradient correction, respectively.

Using identical scan parameters apart from a TR of 6000 ms, an asym-pvc scan with an m_1_ value of 0.1 s/mm was performed with a parallel imaging factor of *R* = 2 and *b*-values of [0, 600, 600] s/mm^2^, with each of the *b* = 600 s/mm^2^ acquisitions using a different waveform design for inducing *m*_1_. Waveform 1 is the design of the lower *b*-values, with a separate pgse gradient added on top of the existing asym-vc gradient to increase the *m*_1_. Waveform 2 is the design of the higher *b*-value, meaning that *m*_1_ is added by changing the relative lengths of the gradient lobes of the asym-vc gradients. The above experiment was done to validate that both waveform designs for inducing *m*_1_ give the same ADC estimation.

### In vivo* measurements*

In vivo measurements were carried out in 4 healthy volunteers (mean age, 34 ± 6 years) using the built in 12-channel posterior and 16-channel anterior coil. The study was approved by the local ethics commission and all volunteers have consented for their participation in the study.

Two slices of the liver were acquired in the coronal plane with a FoV of 240 × 312 mm^2^, a voxel size of 3 × 3 × 6 mm^3^, parallel imaging acceleration factor *R* = 3, effective bandwidth over phase encode direction = 53.3 Hz/pixel, number of packages = 1, *b*-values of [200, 600] s/mm^2^ and corresponding averages of [3, 4]. The *b*-value of 200 s/mm^2^ was chosen to minimize any perfusion signal contributions to the ADC estimation. To reduce echo time for better SNR, diffusion encoding directions [− 0.5 − 1 − 1], [1 0.5 − 1], [1 − 1 0.5] were used. These diffusion encoding directions (which shall be referred to from now on as the overplus diffusion directions) were used for all four subjects, giving echo times of 53/80/80/90 ms for pgse, asym-vc, asym-pvc and sym-vc respectively. The TR was determined by the breathing and cardiac cycle of each volunteer, and was in the range of approximately 3000–6000 ms.

For three out of the four subjects, scans with diffusion encoding along the RO, PE and SS directions (magnet diffusion directions) were additionally performed, giving echo times of 60/88/88/94 ms for pgse, asym-vc, asym-pvc and sym-vc, respectively. This was not done for the fourth subject due to time constraints and the fact that this subject had lower liver signal, meaning that the increased echo time would have reduced the image quality.

Scans with both cardiac and respiratory triggering (double triggering), as well as scans with only respiratory triggering were performed for both sets of diffusion directions (magnet and overplus). The imaging parameters were identical between the double-triggered and respiratory-triggered scans.

ADC maps were generated, and subtraction maps were computed from subtracting the ADC maps without any gradient nonlinearity correction from the ADC maps with gradient nonlinearity correction. For the ROI analysis, ROIs were drawn on one slice of the pgse ADC map from the respiratory-triggered scans. These ROIs were then propagated to the subtraction maps for each diffusion encoding waveform. One of the ROIs was drawn in the left liver lobe in a region with an overestimated ADC due to motion corruption, taking care to avoid large vessels. This ROI was chosen to investigate interactive effects of both motion and gradient nonlinearity on the ADC estimation. The other ROI was drawn close to the right inferior side of the liver.

### Correction maps

Figure 2 shows correction maps of the gradient nonlinearity correction for the RO, PE and SS diffusion directions. The correction maps calculated from the gradient field spherical harmonics for each diffusion direction were equal between the different diffusion encoding waveforms.

## Results

### Phantom results

**Fig. 3:**
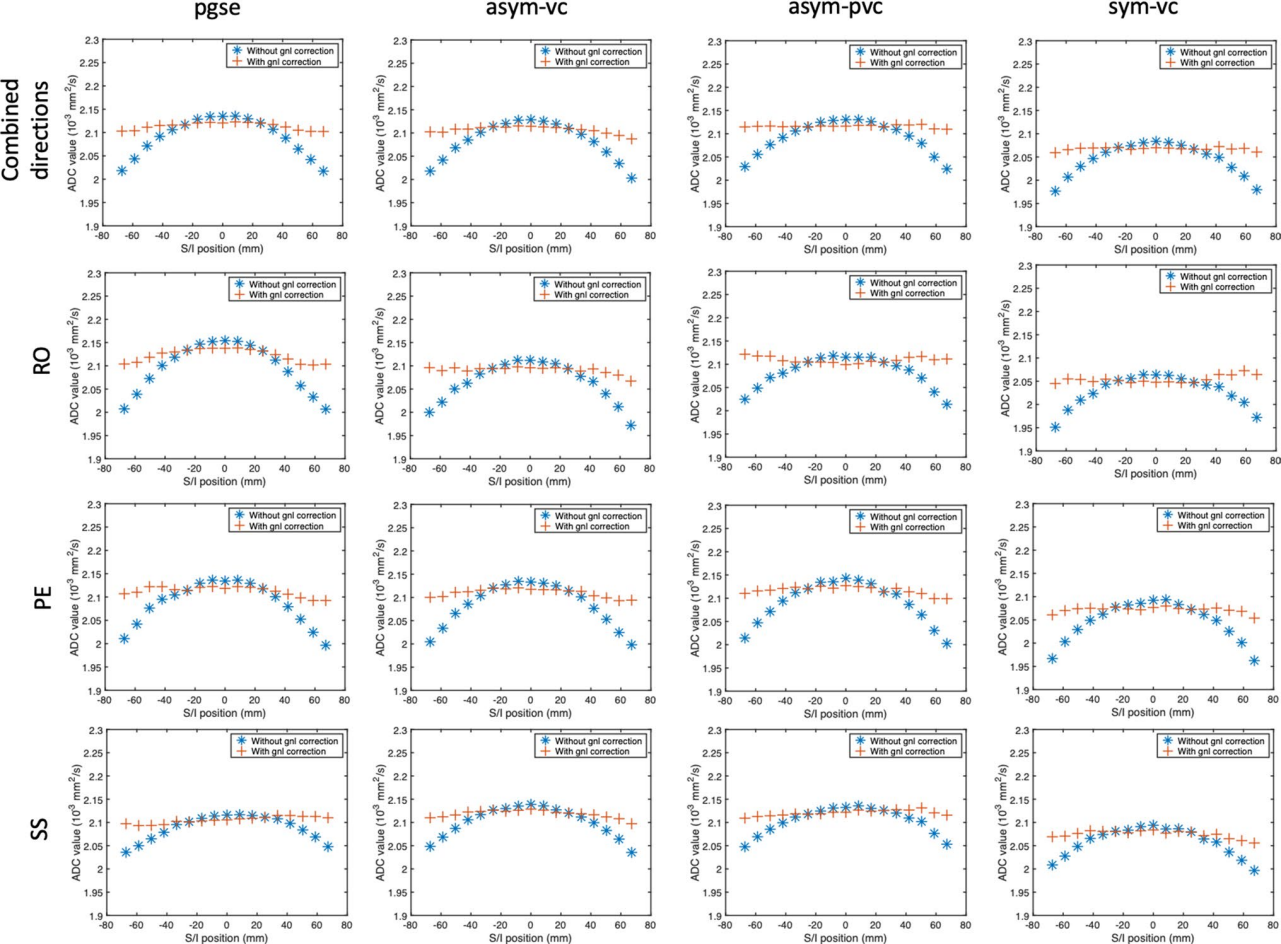
Gradient nonlinearity along the S/I direction. The curve of the non-corrected ADC for an ROI drawn in the center of the phantom shows the characteristic gradient-nonlinearity-induced shape. After correction, this bias is considerably reduced for all waveforms

Figure 3 shows the results from the phantom experiment in which slices were acquired in the S/I direction (gradient nonlinearity in the S/I direction). Without the gradient nonlinearity correction, along the S/I direction, the average ADCs exhibit the characteristic gradient nonlinearity curve, in which a local maximum or minimum is at the isocenter, and the mean ADC increases or decreases symmetrically away from the isocenter. After the gradient nonlinearity correction, the pgse waveform yields minimal variation of ADC for each diffusion direction. Before gradient nonlinearity correction, the standard deviation of the ADC (calculated from all directions combined) across all slices is 4.36 × 10^–5^ mm^2^/s. After gradient nonlinearity correction, this is 1.02 × 10^–5^ mm^2^/s. The range of the ADC values for pgse over a distance of ± 67.2 mm from isocenter is 1.29 × 10^–4^ mm^2^/s before gnl correction, and 0.32 × 10^–4^ mm^2^/s after gnl correction. Similar to the pgse waveform, the asym-vc, asym-pvc and sym-vc waveforms also show the characteristic ADC variation when no gradient nonlinearity correction is performed. With the gradient nonlinearity correction, the curves were flattened for all waveforms. The asym-vc waveform has a standard deviation of ADC (calculated from all directions combined) across all slices of 4.08 × 10^–5^ mm^2^/s before gnl correction, and 0.75 × 10^–5^ mm^2^/s after gnl correction. The range of the ADC values for asym-vc over a distance of ± 67.2 mm from isocenter is 1.22 × 10^–4^ mm^2^/s before gnl correction, and 0.24 × 10^–4^ mm^2^/s after gnl correction. The sym-vc waveform has a standard deviation of ADC (calculated from all directions combined) across all slices of 3.16 × 10^–5^ mm^2^/s before gnl correction, and 0.60 × 10^–5^ mm^2^/s after gnl correction. The range of the ADC values for sym-vc over a distance of ± 67.2 mm from isocenter is 1.04 × 10^–4^ mm^2^/s before gnl correction, and 0.22 × 10^–4^ mm^2^/s after gnl correction. The asym-pvc waveform shows a slight overcompensation in the RO direction, as the curve looks inverted compared to the case without gradient nonlinearity correction; however, the variation in ADC is still reduced considerably. This waveform has a standard deviation of ADC (calculated from all directions combined) across all slices of 3.54 × 10^–5^ mm^2^/s before gnl correction, and 0.26 × 10^–5^ mm^2^/s after gnl correction. The range of the ADC values for asym-pvc over a distance of ± 67.2 mm from isocenter is 1.07 × 10^–4^ mm^2^/s before gnl correction, and 0.11 × 10^–4^ mm^2^/s after gnl correction. For all waveforms, with or without gradient nonlinearity correction, any observable dependence of the ADC variation on the diffusion encoding direction is small. The results from the phantom experiment in which slices were acquired in the R/L direction (gradient nonlinearity in the R/L direction) are shown in the supplementary material Fig. S2.

### Concomitant gradient phantom experiment

A simulation of the effect of concomitant gradients on the ADC value in a phantom is given in the Supplementary Material. The difference in ADC for a range of distances from the isocenter is given in the Supplementary Material Fig. S4.

**Fig. 4:**
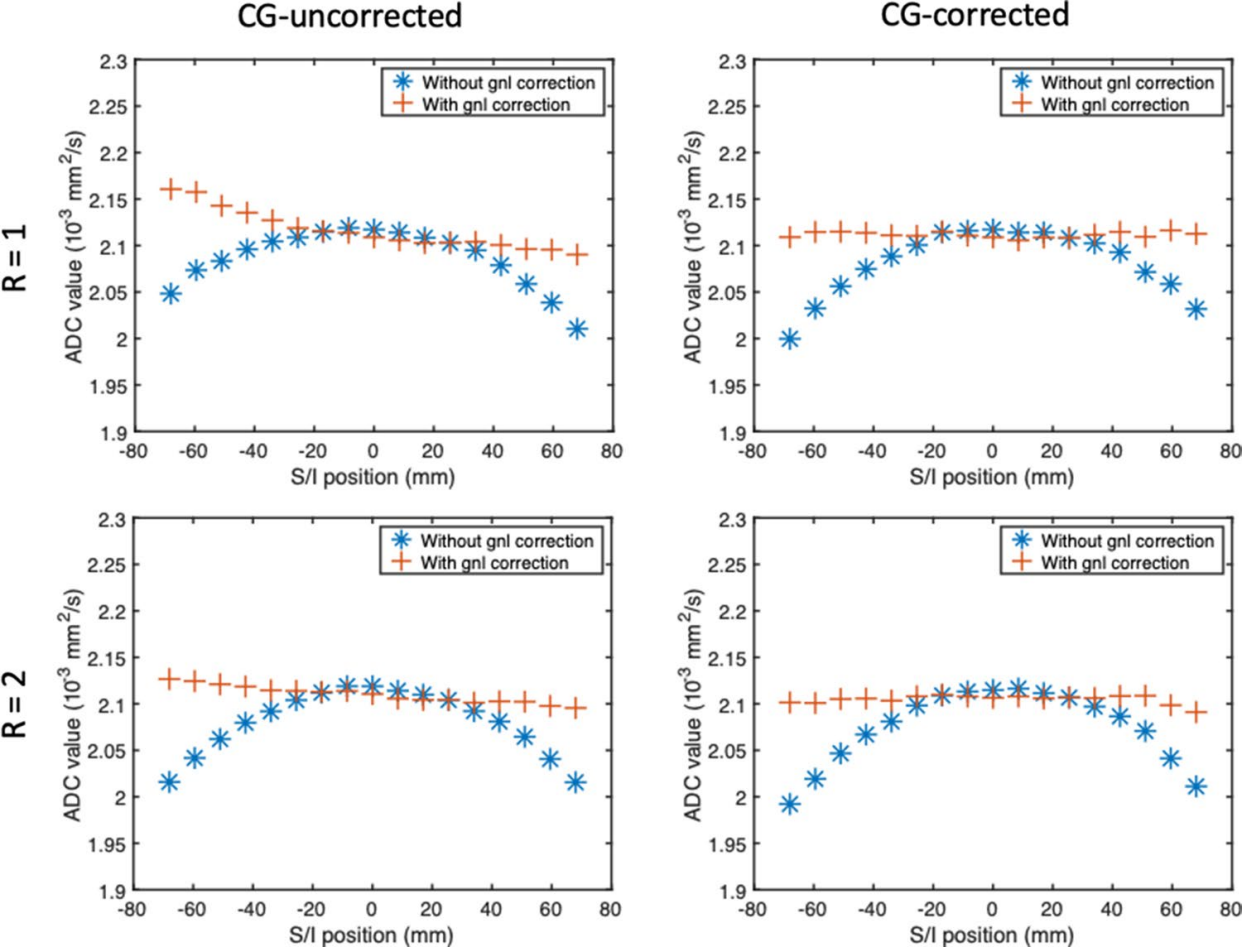
asym-vc waveform with and without concomitant gradient correction. When no parallel imaging factor is used (*R* = 1), there is a considerable overestimation of the ADC on one side of the CG-uncorrected curve after gnl correction. When a parallel imaging factor of 2 (*R* = 2) is used, the number of acquired *k*-space lines is reduced in comparison to when no parallel imaging is used. Therefore, the time interval available for diffusion encoding after the refocusing pulse is closer to the time interval before the refocusing pulse, meaning that the diffusion encoding waveforms before and after the refocusing pulse are more symmetrical, and the effect of concomitant gradients is therefore not as large. The CG-corrected waveform has a fairly constant ADC across the FoV after gnl correction

Figure 4 shows the phantom results for the asym-vc waveform with and without concomitant gradient correction for two different parallel imaging factors. When no parallel imaging is used, the difference in ADC between a slice approximately 60 mm away from isocentre and a slice at isocentre was approximately 4.9 × 10^–5^ s/mm^2^. When a parallel imaging factor of 2 was used, this ADC difference was 1.4 × 10^–5^ s/mm^2^. When no parallel imaging was used, the standard deviation of the ADC of the asym-vc with concomitant correction was 3.55 × 10^–5^ s/mm^2^ before gnl correction, and 0.30 × 10^–5^ s/mm^2^ after gnl correction. The standard deviation of the ADC of the asym-vc without concomitant correction was 3.14 × 10^–5^ s/mm^2^ before gnl correction, and 2.14 × 10^–5^ s/mm^2^ after gnl correction, indicating that the CG correction is performing well. When a parallel imaging factor of 2 was used, meaning that the waveform was less asymmetric and therefore would not have as severe concomitant gradient effects, the standard deviation of the ADC of the asym-vc without concomitant correction was 3.48 × 10^–5^ s/mm^2^ before gnl correction, and 0.93 × 10^–5^ s/mm^2^ after gnl correction. The standard deviation of the ADC of the asym-vc with concomitant correction was 3.99 × 10^–5^ s/mm^2^ before gnl correction, and 0.47 × 10^–5^ s/mm^2^ after gnl correction, again remaining stable.

### asym-pvc waveform comparison

**Fig. 5:**
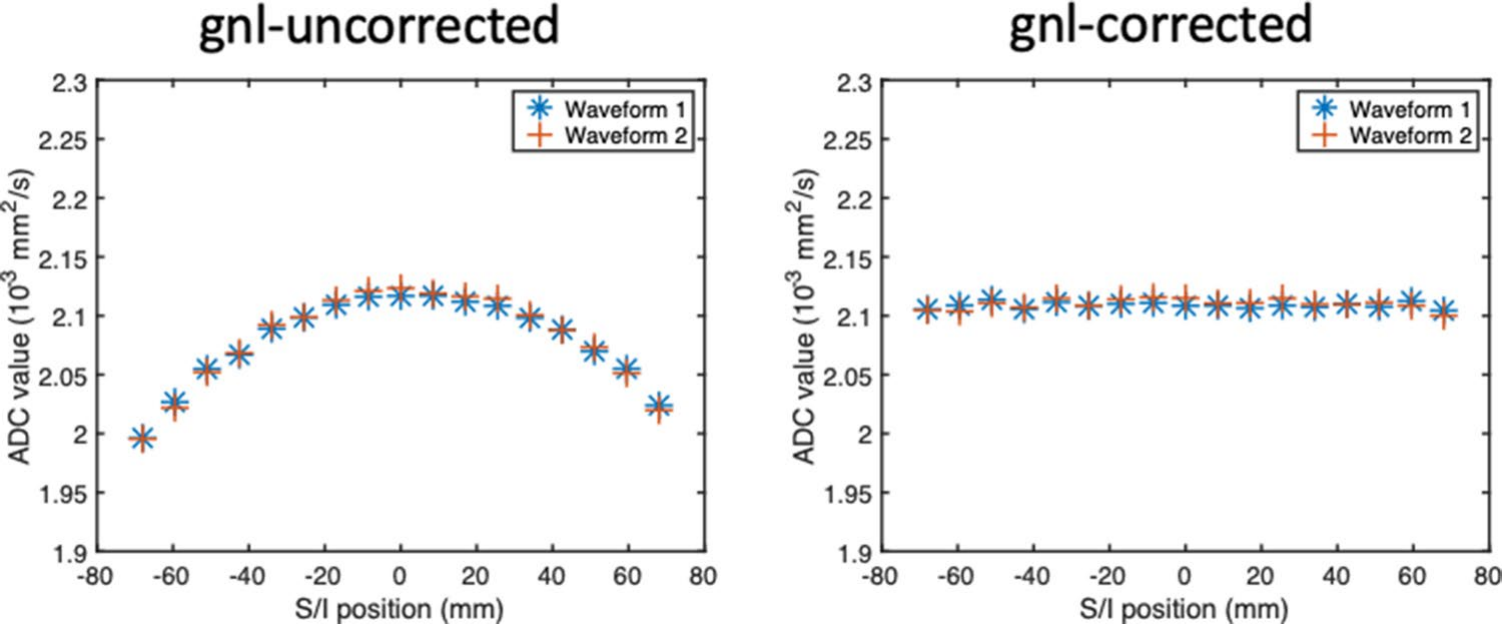
asym-pvc waveform (*m*_1_ = 0.1 s/mm) with ADC calculated from both of the waveform designs. Waveform 1 is the design of the lower *b*-values, with a separate pgse gradient added on top of the existing asym-vc gradient to increase the *m*_1_. Waveform 2 is the design of the higher *b*-value, meaning that *m*_1_ is added by changing the relative lengths of the gradient lobes of the asym-vc gradients. Both waveforms give almost identical ADC values

Figure 5 shows the phantom results for the comparison between the two different waveforms used to make up asym-pvc. The maximum difference in ADC between the two waveforms at the same slice was 0.66 × 10^–5^ s/mm^2^ before gnl correction, and 0.65 × 10^–5^ s/mm^2^ after gnl correction.

### In vivo* results*

**Fig. 6:**
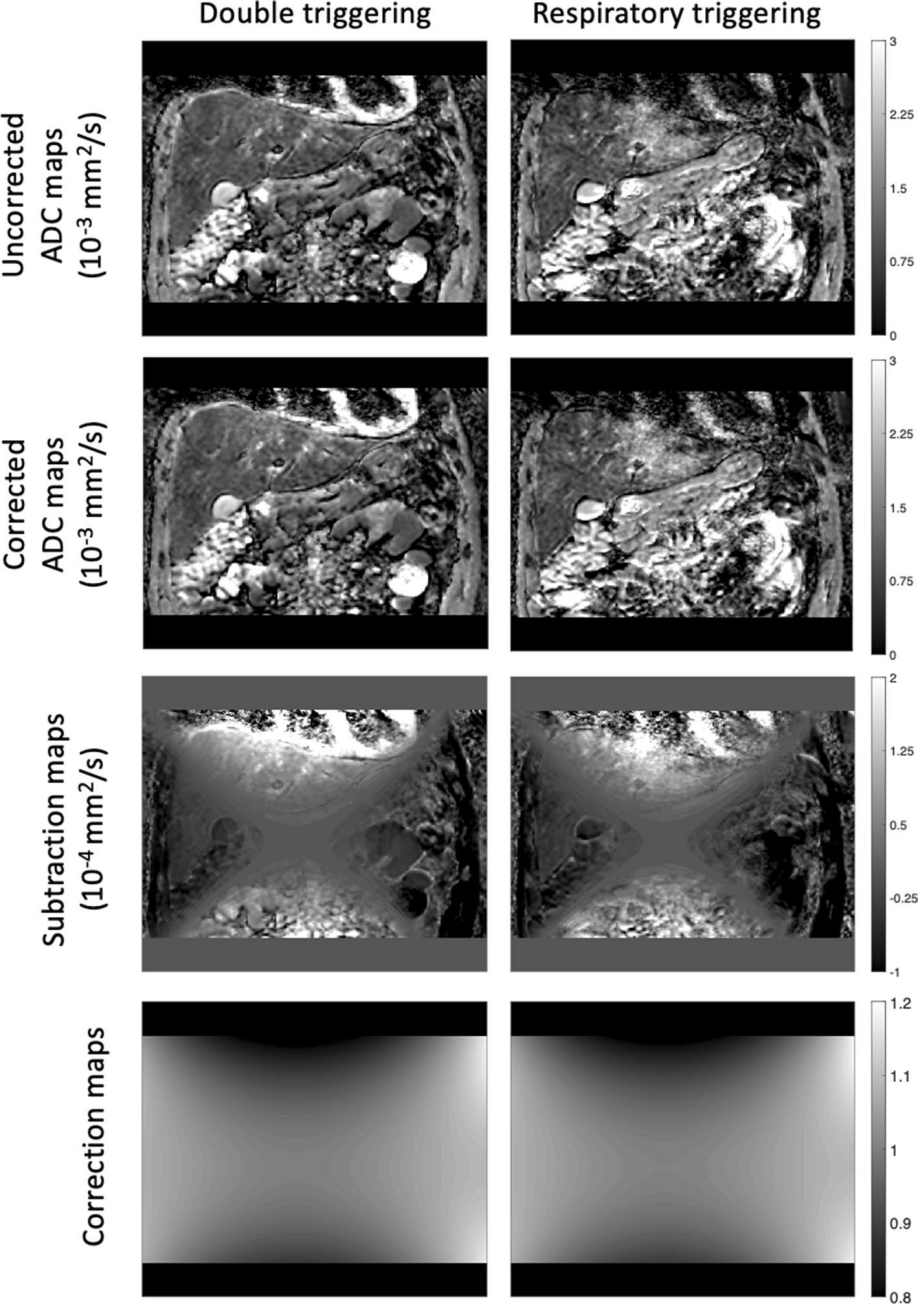
Uncorrected ADC maps, corrected ADC maps, subtraction maps (calculated by subtracting the uncorrected ADC map from the corrected ADC map) and correction maps for the pgse waveform with double triggering and only respiratory triggering for one volunteer. The respiratory-triggered case shows a clear overestimation of the ADC in the left liver lobe. The subtraction map shows a positive value in this region, highlighting an interaction between motion effects and gnl correction. The gnl correction will increase an already overestimated ADC in this region, as shown in the corrected ADC maps

Figure 6 shows the uncorrected ADC maps, corrected ADC maps, subtraction maps and correction maps for two different scans performed with the pgse waveform, one with double cardiac and respiratory triggering, and one with only respiratory triggering. It is clear that in the left liver lobe region when only respiratory triggering is used, the ADC is overestimated. In the subtraction maps, the left liver lobe region also has high values (with a mean ADC difference of 1.4 × 10^–5^ mm^2^/s for the respiratory-triggered scans, and 1.0 × 10^–5^ mm^2^/s for the double-triggered scans), indicating that the gradient nonlinearity correction has increased the motion-induced overestimated ADC further. ADC maps and subtraction maps from two volunteers are shown in the supplementary material Fig. S5.

**Fig. 7:**
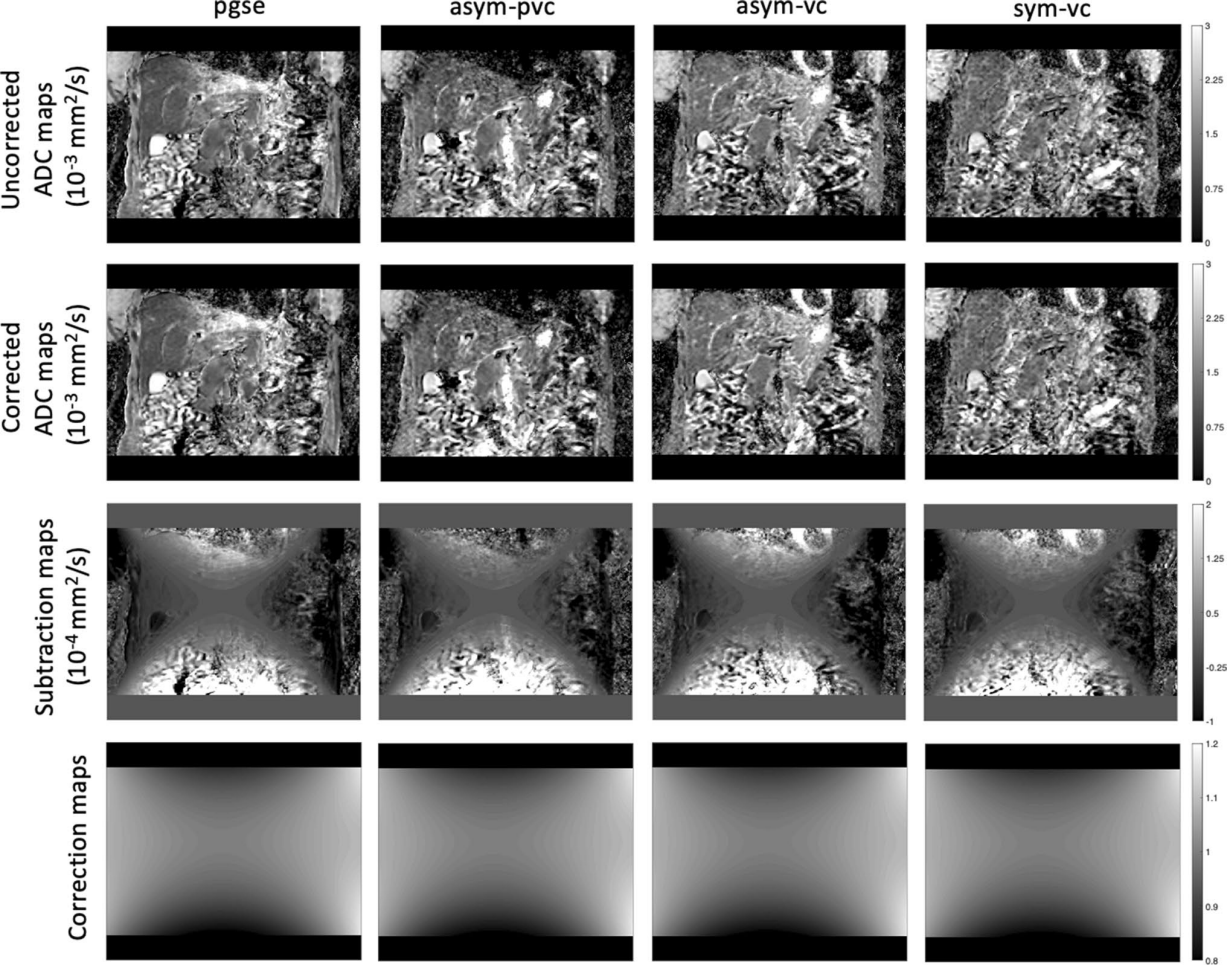
Uncorrected ADC maps, corrected ADC maps, subtraction maps and correction maps for all waveforms. The data were acquired with diffusion encoding along the overplus directions. The correction maps were the same for all waveforms. The uncorrected ADC maps for the motion-compensated waveforms do not show as large of an ADC overestimation in the left liver lobe when compared with pgse. The subtraction maps therefore do not show as large of a difference between the gnl corrected and non-corrected ADC maps, apart from in regions where there are large vessels. The corrected ADC maps for the motion-compensated waveforms also do not show as large of an ADC overestimation in the left liver lobe when compared with pgse

Figure 7 shows the uncorrected ADC maps, corrected ADC maps, subtraction maps and correction maps for all waveforms in which only respiratory triggering was used. The diffusion encoding directions were the overplus directions for the first volunteer, and RO, PE and SS for the second volunteer. The ADC in the left liver lobe when the pgse waveform was used is again overestimated. The subtraction map shows that the gradient nonlinearity correction has increased the motion-induced overestimated ADC even further. The motion-compensation capability of the asym-pvc, asym-vc and sym-vc waveforms reduces the motion-induced overestimation of ADC at the cost of lower SNR, compared to when the pgse waveform was used. Subtraction maps show lower values in the left liver lobe for asym-pvc, asym-vc and sym-vc waveforms compared to the pgse waveform. Since bright vessels will also have a high ADC, this is also further increased by the gradient nonlinearity correction in certain regions, depending on the correction map. This effect can also be seen in the subtraction maps. The effect of gnl correction on overestimated ADC is explained further in the supplementary material Fig. S3. ADC maps and subtraction maps from two volunteers are shown in the supplementary material Fig. S6.

**Fig. 8:**
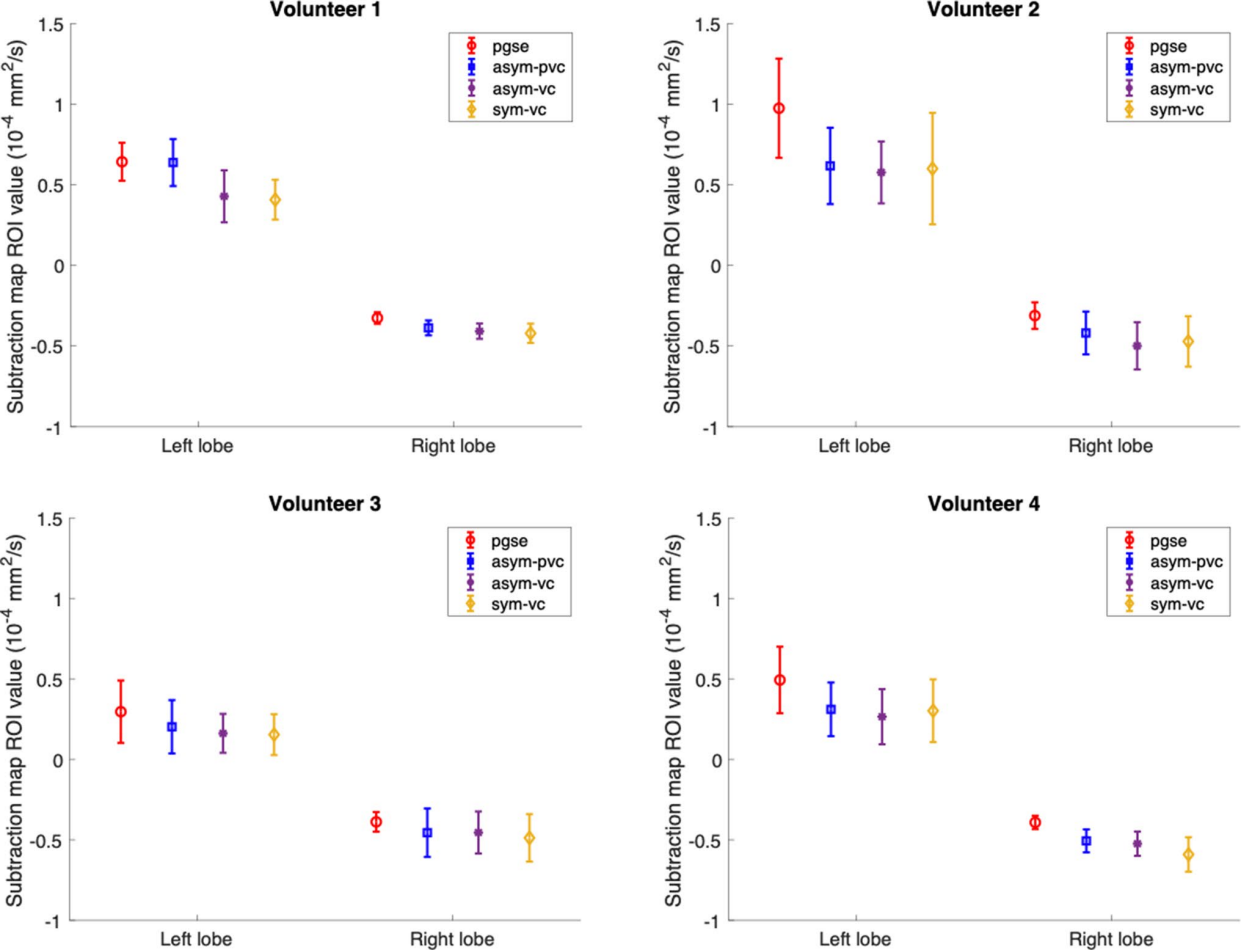
Subtraction map ROI analysis for all subjects in which an ROI was drawn in the left liver lobe in a region that had been corrupted by motion and another ROI was drawn in the right inferior region of the liver. In the left lobe region, the motion-compensated waveforms have a lower mean value in the subtraction map ROI, possibly as a result of the decreased sensitivity to motion. The differences between the waveforms in the right liver lobe region, which is not heavily affected by motion, are typically smaller

Figure 8 shows the ROI analysis performed on the subtraction maps for all subjects for all waveforms. The plots show the mean value of the subtraction map over the ROI, and the error bars show the standard deviation over the ROI. In all subjects, the asym-pvc, asym-vc and sym-vc waveforms had a lower mean subtraction map value in the left liver lobe region compared to the pgse waveform. In the right liver lobe region, the differences between all waveforms are typically smaller.

## Discussion

The present work applies the gradient nonlinearity correction using the spherical harmonic expansion of the gradient coil field [33] to different motion-compensated diffusion encoding waveforms. Despite known disadvantages such as the prolonged echo time and reduced SNR, occurrence of bright vessel signal, concomitant effects if asymmetrical waveforms are used, motion-compensated diffusion encoding waveforms remain an alternative approach to more accurate ADC quantification. The gradient nonlinearity correction has, to the best of our knowledge, only been applied to the standard monopolar pgse diffusion encoding waveform and the present work, therefore, showed the feasibility of using the gradient nonlinearity correction with motion-compensated diffusion encoding waveforms.

Although not the main focus of the present study, the present work also proposes methods for online computation of near-TE-optimal motion-compensated and partial motion-compensated waveforms with concomitant field correction, allowing flexibility in protocol optimization [37, 38]. The presently employed methods utilize the knowledge from the offline TE-optimized gradient waveform design as proposed by Peña-Nogales et al. [19]. However, the implementation of the gradient nonlinearity correction was found to be applicable to different motion-compensated waveform designs, and should also be applicable to any diffusion encoding waveform designed offline or on-the-fly as in [37, 38]. The asym-pvc achieved partial velocity compensation by adjusting the lengths of the lobes for the maximum *b*-value, and by adding small pgse gradients to the asym-vc waveform for the lower *b*-values. The difference in design between the *b*-values was done to mimic the design of Zhang et al. [15] and for ease of implementation.

Figure 2 shows the correction maps for the RO, PE and SS directions. Since the employed correction procedure holds for a general gradient waveform in which the polarity is reversed at TE/2 [33, 39] (to implicitly account for the effect of the spin echo RF pulse [40]), the correction maps were the same for all waveforms.

The phantom results show that the gradient nonlinearity correction can considerably reduce ADC bias for all waveforms. All waveforms showed some residual fluctuation after correction, which can be attributed to higher order gradient nonlinearity effects, or other factors such as eddy currents or residual concomitant gradient effects and is a subject for further investigation. The asym-pvc waveform showed a slight overcompensation in the RO direction, as the curve looks inverted compared to the case without gradient nonlinearity correction. This could be a result of residual concomitant gradient effects. When the current nonlinear gradient correction method is employed, similar levels of residual fluctuation of ADC values were observed across all waveforms, suggesting that the employed method can correct for gradient nonlinearity just as well in the case of the more complicated motion-compensated waveforms.

The gnl correction can be affected by non-gnl related ADC errors. Evidence of such interactions between gnl correction and non-gnl related ADC errors was shown in Figs. 6 and 8 in the left liver lobe and regions with bright vessels.

The phantom experiment for the asym-vc concomitant gradient correction in Fig. 4 showed that even when no parallel imaging factor was used, therefore making the waveform more asymmetric and increasing the impact of the concomitant fields, the CG-corrected waveform remained stable. The CG-uncorrected waveform showed larger deviations in ADC away from isocentre when no parallel imaging factor was used. When a parallel imaging factor of 2 was used, the variation in ADC was lower; however, it was still larger than the CG-corrected waveform, indicating that the concomitant correction performed well.

The phantom experiment for the two different waveform designs for asym-pvc in Fig. 5 showed that the ADC calculated from both waveform designs was almost identical, justifying the usage of using different designs for the lower and higher *b*-values in the asym-pvc waveform.

The subtraction map ROI analysis in Fig. 8 showed that for all subjects, the pgse waveform had a higher subtraction map value in the left liver lobe in a region which has overestimated ADC due to motion. The higher subtraction map value highlights the interaction between the gradient nonlinearity correction and ADC overestimation. In the right liver lobe region, the differences between the pgse waveform and the motion-compensated waveforms were smaller, as this is a region that is not as heavily affected by motion. The asym-pvc waveform also typically had a higher subtraction map value in the left liver lobe than the asym-vc waveform, which could be due to the fact that the increase in m_1_ also increases sensitivity to motion. In the right liver lobe region, the motion-compensated waveforms also had a larger standard deviation over the ROI in the subtraction map than pgse, which could be explained by the lower SNR and bright vessel signal. Therefore, the presented results suggest that to get accurate ADC quantification, one has to take gradient nonlinearity, motion sensitivity, vessel signal suppression and the impact of these on each other into account.

The present work has some limitations. First, the phantom measurements were performed without any temperature control. In Malyarenko et al. [33], an ice water phantom was used to keep the temperature constant throughout the experiment. Second, only a distance of ± 67.2 mm from isocentre was measured with the presently used phantom. Despite these limitations, the pgse results without the gradient nonlinearity correction showed the characteristic curve of ADC bias, which was flattened by the gradient nonlinearity correction. Third, only four healthy volunteers were scanned in the present work. Further work would be required to fully characterize the interaction between gradient nonlinearity-induced and motion-induced bias in liver ADC quantification of subjects with different motion patterns and liver ADC variations.

## Conclusion

The gradient nonlinearity correction based on the spherical harmonic expansion of the gradient coil field was used in conjunction with different motion-compensated waveforms, thereby showing the feasibility of the usage of the gradient nonlinearity correction with gradient waveforms other than the standard monopolar pgse diffusion encoding waveform. The phantom results showed a reduction in ADC bias in all waveforms after the gradient nonlinearity correction was applied. The in vivo results showed an interaction between the gradient nonlinearity correction, ADC overestimation in the left liver lobe due to motion, and bright vessel signal from the motion-compensated waveforms. This suggests that to get accurate ADC quantification, one has to take gradient nonlinearity, motion sensitivity, vessel signal suppression and the impact of these on each other into account.

## Supplementary Information

Below is the link to the electronic supplementary material.

Supplementary file1 (DOCX 2661 KB)

## Abbreviations

DWI: Diffusion-weighted imaging
ADC: Apparent diffusion coefficient
gnl: Gradient nonlinearity
EPI: Echo planar imaging
ODGD: Optimized diffusion-weighting gradient waveform design
pgse: Pulsed gradient spin echo
sym-vc: Symmetric velocity compensated
asym-vc: Asymmetric velocity compensated
asym-pvc: Asymmetric partial velocity compensated
