# Supplementary material for: Gradient nonlinearity correction in liver DWI using motion-compensated diffusion encoding waveforms
Source: MAGMA. 2021 Dec 11;35(5):827–41. doi: 10.1007/s10334-021-00981-6 (PMC9463296; doi:10.1007/s10334-021-00981-6)
Supplement: Supplementary file 1 — Supplementary file1 (DOCX 2661 KB) [file 10334_2021_981_MOESM1_ESM.docx]

**WAVEFORM DESIGN**

The differences between the asym-vc, Convex optimized diffusion encoding (CODE) [1] and Optimized Diffusion-Weighting Gradient Waveform Design (ODGD) [2] waveforms are highlighted here.

**Main constraints**

For all waveforms, the diffusion gradient is set to 0 during the excitation pulse, refocusing pulse and the readout.

$$G\left( T_{RF-90} \right)=0, G\left( T_{RF-180} \right)=0, G\left( T_{EPI} \right)=0$$

All waveforms have the same hardware constraints, namely that the maximum gradient and slew rate cannot not exceed their respective values. The ODGD waveform takes the general equation for the b-value:

$$b= \gamma^{2}\int_{0}^{T_{Diff}} {F(t)}^{2}dt, F\left( t \right)= \int_{0}^{t} G\left( \tau\right)d\tau,$$

and maximizes this for a given TE.

In the CODE formulation, instead of optimizing the quadratic b-value equation, the linear term

$$\beta=\int_{0}^{T_{Diff}} F\left( t \right)dt$$

is maximized.

For the asym-vc waveform, the b-value equation is expressed in terms of the gradient lobe timings, which is then input into the scanner software environment and the echo time is optimized for a given b-value.

**Consideration of concomitant gradient effects**

The ODGD waveform uses the equation for the concomitant gradient term as one of the constraints. The CODE waveform linearly approximates the phase variations from the concomitant fields and adds a gradient magnitude offset to compensate. The asym-vc waveform uses the concomitant field equation as one of the constraints and adds the length of the duration of the slope between the first and second gradient lobes as a variable. This slope is then extended when concomitant gradient correction is used.

**Diffusion encoding waveform design and optimization**

In the design of the ODGD and CODE waveforms, no assumptions were made about the number of gradient lobes or how many were before or after the refocusing pulse. The asym-vc waveform is assumed to have 4 lobes, 2 before and 2 after the refocusing pulse. The other constraints for the timing of each lobe are the m_0_, m_1_ and concomitant constraints. The variables to solve for, in terms of the amount of time before and after the refocusing pulse, are the duration of each plateau of each lobe and the slope in between the first and second lobes. The slopes of the other lobes are constant and equal to the maximum slew rate.

The calculation of the asym-pvc waveform for all b-values other than the highest b-value is as follows. Starting from the asym-vc waveform, where each lobe has a pre-defined length, the required strength of the additional pgse gradients for a given m_1_ are calculated. The positions of the additional pgse gradients are shown in Figure S1. The strength of the asym-vc (m_1_ = 0) section of the waveform is incrementally increased until the desired b-value is achieved.

The calculation of the asym-pvc waveform for the highest b-value is as follows. Starting from the asym-vc waveform, the lengths of the first and third lobes are decreased by a small increment, whereas the lengths of the second and fourth lobes are increased by the same amount. The gradient strength is adjusted until the desired b-value is achieved, and the amount of m_1_ given by the new configuration of gradient lobe timings and strength is then calculated. This entire process is repeated until the desired m_1_ is achieved. This process is shown in Figure S1.


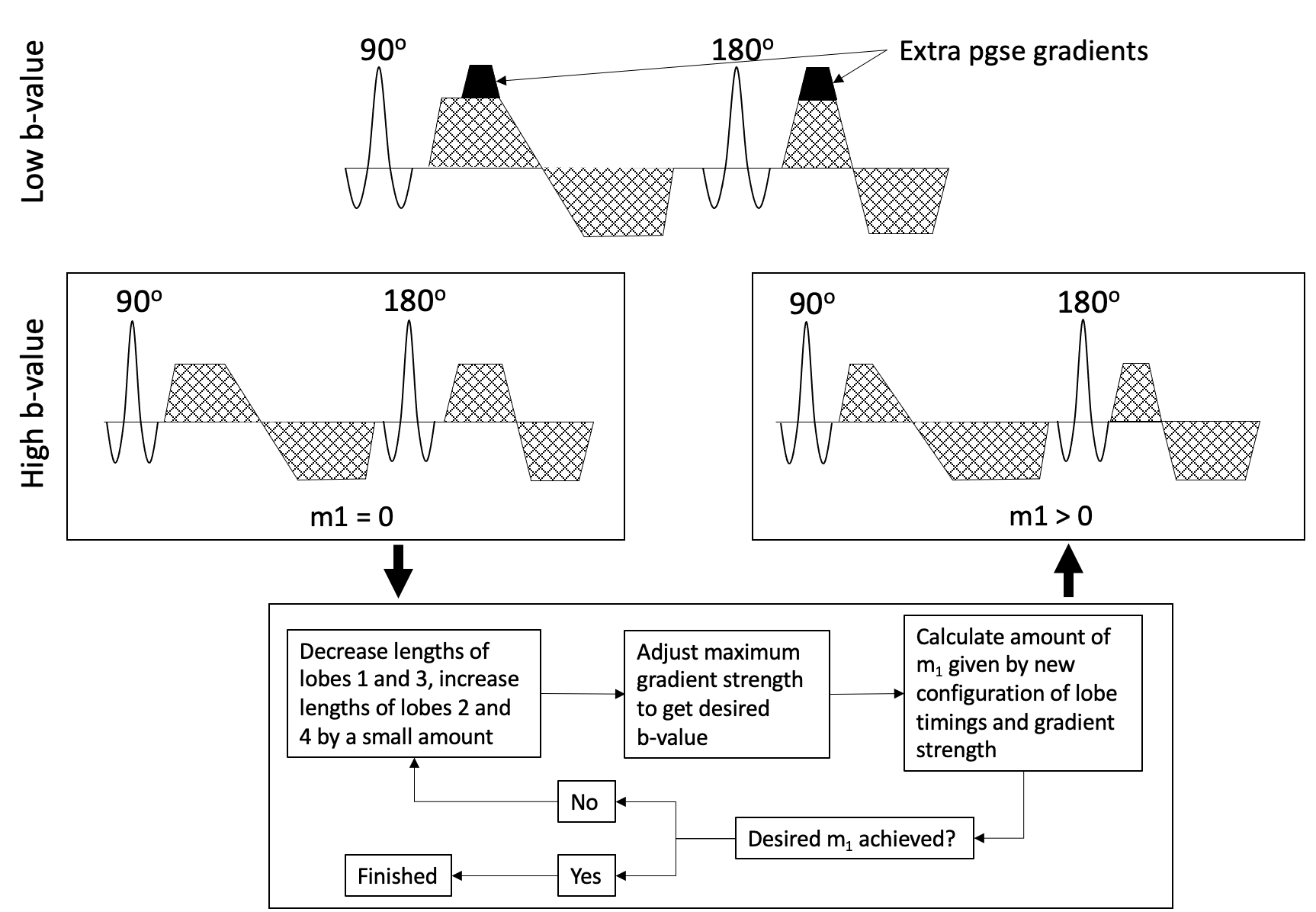


**Fig. S1** Waveform design for the asym-pvc waveform. For all b-values other than the largest b-value, extra pgse gradients are added to the asym-vc waveform in order to achieve the desired m_1_. For the largest b-value, the relative lengths of each gradient lobe are adjusted until the desired m_1_ is achieved

**PHANTOM EXPERIMENT IN R/L DIRECTION**

Phantom validation measurements were performed to assess the performance of the gradient nonlinearity correction with the pgse, sym-vc, asym-vc and asym-pvc waveforms. A cylindrical phantom of length 20cm and diameter 11.5cm containing 31.5 mmol/L of NiCl_2_-6H_2_O was placed inside an extremity coil at isocenter and sagittal slices were acquired with a ss-EPI sequence in the R/L direction to investigate the gradient nonlinearity in the R/L direction.

For all waveforms in the sagittal slice experiment, seventeen slices were acquired with a FoV of 128 x 128 mm^2^, a voxel size of 2 x 2 x 6 mm^3^ and a 2.5 mm slice gap. No parallel imaging was used, bandwidth = 27.2 Hz/pixel. b-values of [0, 600] s/mm^2^ were used for the ADC calculation, with 5 averages per b-value and 3 orthogonal diffusion encoding directions along the readout (RO), phase encoding (PE) and slice selection (SS) directions. The echo times were 75 / 103 / 103 / 116 ms for pgse, asym-vc, asym-pvc and sym-vc respectively. TR = 5000 ms in all cases and no partial Fourier encoding was used for any of the waveforms. An m_1_ value of 0.1 s/mm was used for the asym-pvc waveform.

Figure S2 shows the results from the phantom experiment in which slices were acquired in the R/L direction (gradient nonlinearity in the L/R direction). In comparison to the S/I direction, the curve of the ADC variation when no gradient nonlinearity correction is applied is not as smooth and not as symmetrical about the isocenter. When all diffusion directions are averaged and in the SS direction, there is a clear improvement in the ADC variation for all waveforms. For the PE and RO directions, the variation in ADC further away from isocentre is not as large before gradient nonlinearity correction. For the pgse waveform, when all diffusion directions are averaged, the standard deviation of the ADC over all slices is 1.75x10^-5^ mm^2^/s before gnl correction, and 0.34x10^-5^ mm^2^/s after gnl correction. For the asym-vc waveform, this ADC variation is 1.89x10^-5^ mm^2^/s before gnl correction, and 0.40x10^-5^ mm^2^/s after gnl correction. For the asym-pvc waveform, this ADC variation is 1.64x10^-5^ mm^2^/s before gnl correction, and 0.39x10^-5^ mm^2^/s after gnl correction. For the sym-vc waveform, this ADC variation is 1.88x10^-5^ mm^2^/s before gnl correction, and 0.40x10^-5^ mm^2^/s after gnl correction.


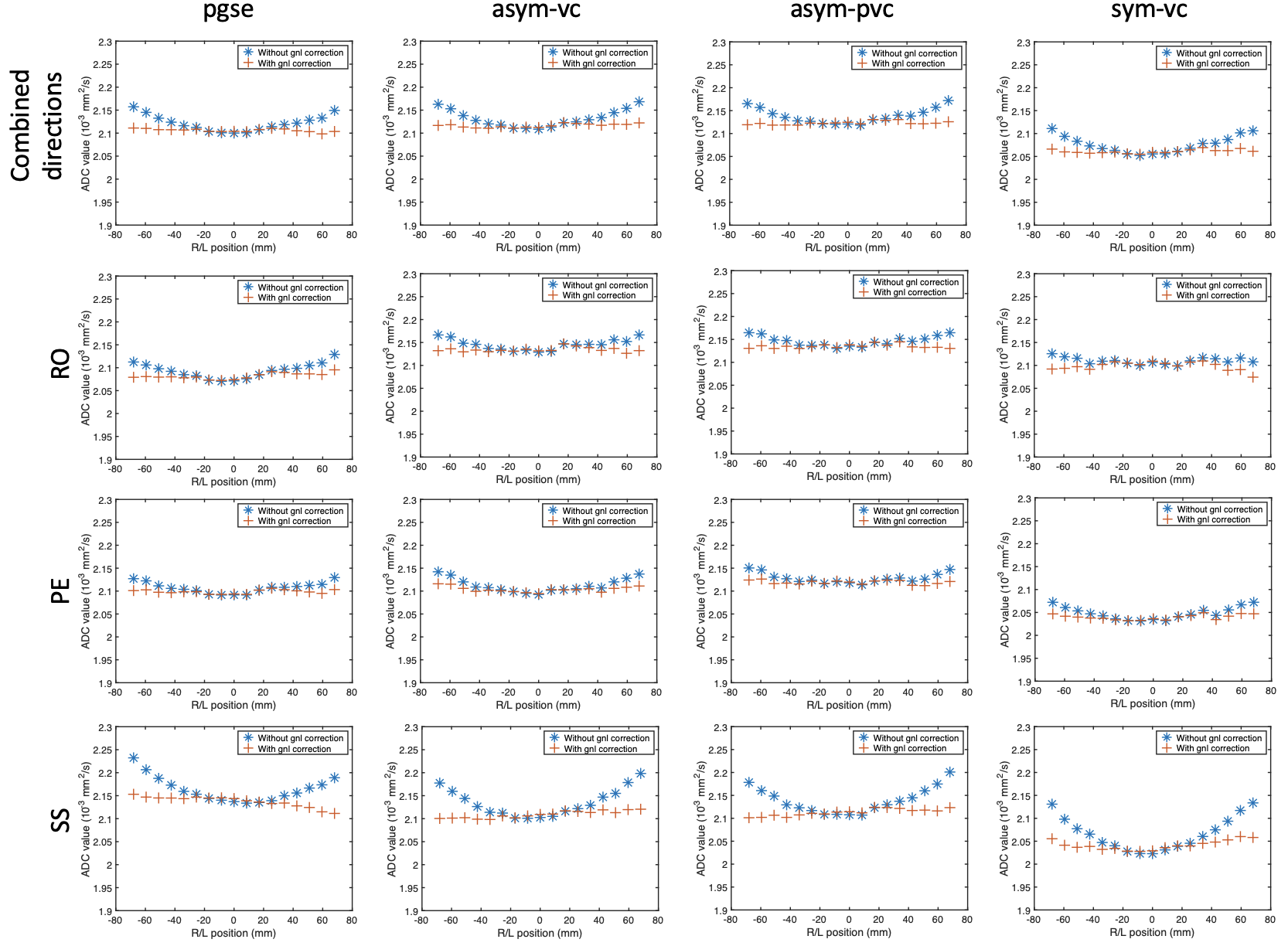


**Fig. S2** Gradient nonlinearity along the R/L direction. Compared to the S/I data, the curves are typically not as smooth, which has an effect of the gradient nonlinearity correction

**EFFECT OF GNL CORRECTION ON OVERESTIMATED ADC**


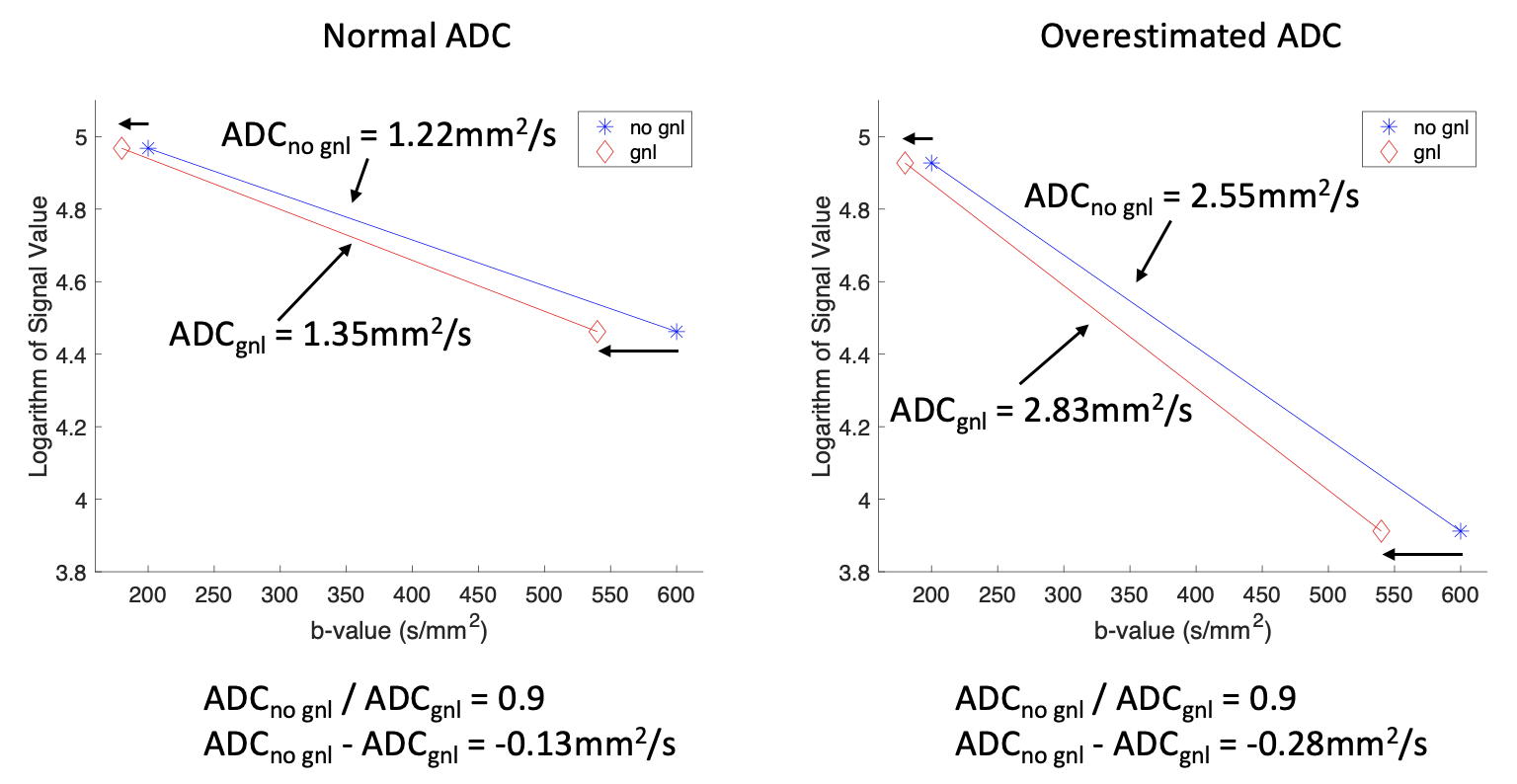


**Fig. S3** Hypothetical example of a point that lies in a region where the b-value is overestimated by 10%. If the ADC is not overestimated, then the ratio of uncorrected to corrected ADC will be 0.9, but the difference will be low. If the ADC is already overestimated (e.g. bias by motion-induced intravoxel signal dephasing), then the ratio of uncorrected to corrected ADC will again be 0.9, but the difference will be larger

**CONCOMITANT GRADIENT SIMULATION**

The asym-vc and asym-pvc waveforms can be designed with and without concomitant gradient correction. The effect of the concomitant gradient correction was simulated. For the sake of simplicity, only the case of through-plane dephasing for an axial slice was considered. The phase $\phi$ due to the concomitant field $B_{C}$over the cumulative duration of the asymmetric diffusion gradients $\tau$ is given by

$\phi\left( x,y,z,\tau\right)= \int_{0}^{\tau} B_{C}\left( x,y,z \right)dt,$( 1 )

Where time t = 0 is taken to be the start of the diffusion encoding waveform. From Baron et al [3], the signal for an arbitrary slice orientation, assuming uniform magnetization across the slice, is given by

$S\left( r_{F}{,r}_{P},r_{S,0} \right)= S_{0}\left( r_{F}{,r}_{P},r_{S,0} \right)\cdot\frac{1}{w} \int_{r_{S,0}-w/2}^{r_{S,0}+w/2} e^{-i\gamma\int_{0}^{\tau} B_{C}\left( x,y,z \right)dt}dr_{S}$, ( 2 )

where $r_{F}{,r}_{P}$and $r_{S,0}$are the distance along the frequency encode direction, the distance along the phase encode direction and the position of the centre of the slice respectively. $S_{0}$ is the signal received if concomitant fields are not present and $w$ is the slice thickness. Assuming a single diffusion gradient in either the x or y direction, a Taylor expansion (a more detailed derivation is given in Bernstein et al [4]) gives the phase accumulation coefficient $A$ due to the concomitant field to be

$A= \frac{\gamma}{2B_{0}}\int_{0}^{\tau} [(G_{x}^{2}\left( t \right)+G_{y}^{2}\left( t \right))_{\tau_{1}}-(G_{x}^{2}\left( t \right)+G_{y}^{2}\left( t \right))_{\tau_{2}}] dt,$( 3 )

as all cross terms will be zero. $G_{x}$ and $G_{y}$ are the gradient strengths in the x and y directions, respectively, and $\tau_{1}$ and $\tau_{2}$ are the times before and after the refocusing pulse, respectively. Again from Baron et al [3], the change in ADC due to through-plane dephasing is given by

$\delta ADC\left( x,y \right)= -ln\left[ sinc\left( A*z_{s}*w \right) \right] ,$( 4 )

where $z_{s}$ is the distance of the slice from isocenter.

By inserting Equation 3 into Equation 4 and taking the gradient timings from the scanner, the change in ADC due to concomitant fields was calculated.

By taking the gradient timings from the scanner, using a slice thickness of 6 mm and taking the z distance from the isocentre to be 60mm, the difference in ADC due to concomitant fields was calculated from Equation 8 to be 1.9x10-6 s/mm2.


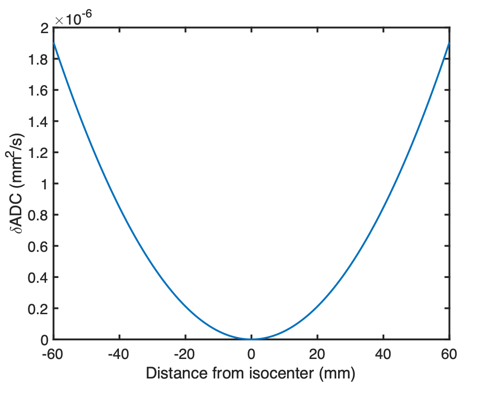


**Fig. S4** Change in ADC from through-plane dephasing due to concomitant fields for a range of distances from the isocenter. The asym-vc waveform was used. From Equation 9 in the main text, there is a $sinc$ function relationship between $\delta ADC$ and the distance from isocenter. The slice thickness was taken to be 6 mm and the diffusion gradient strengths and timings were taken from the scanner. The *b*-values used for the $\delta ADC$ calculation were b = 0 s/mm^2^ and b = 600 s/mm^2^ and the diffusion direction was the *x*-direction (in scanner coordinates).

**EFFECT OF GNL CORRECTION ON LEFT LIVER LOBE**


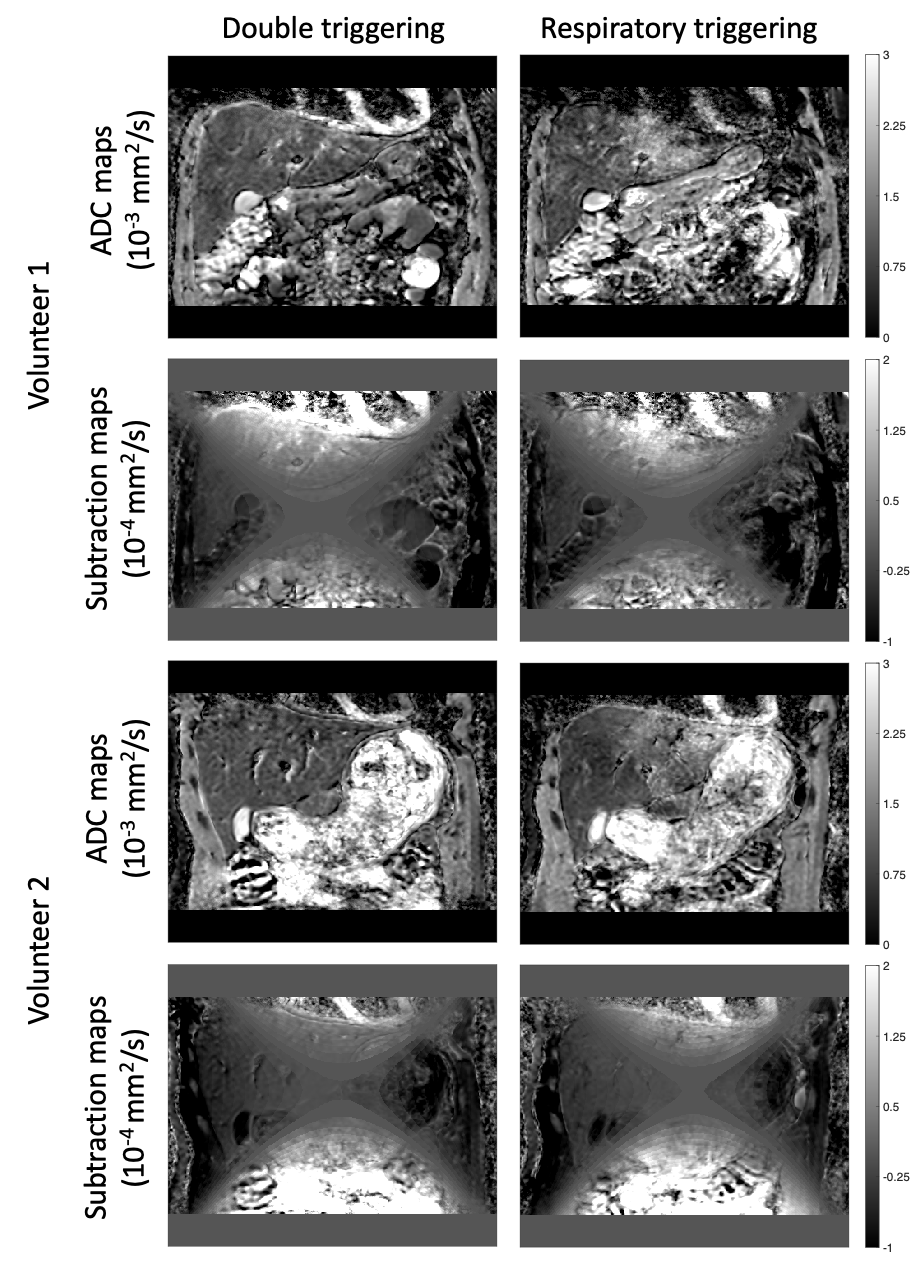


**Fig. S5** ADC maps and subtraction maps (calculated by subtracting the uncorrected ADC map from the corrected ADC map) for the pgse waveform with double triggering and only respiratory triggering. The respiratory triggered case shows a clear overestimation of the ADC in the left liver lobe. The subtraction map shows a positive value in this region, highlighting an interaction between motion effects and gnl correction. The gnl correction will increase an already overestimated ADC in this region

**EFFECT OF GNL CORRECTION ON MOTION COMPENSATED WAVEFORMS**


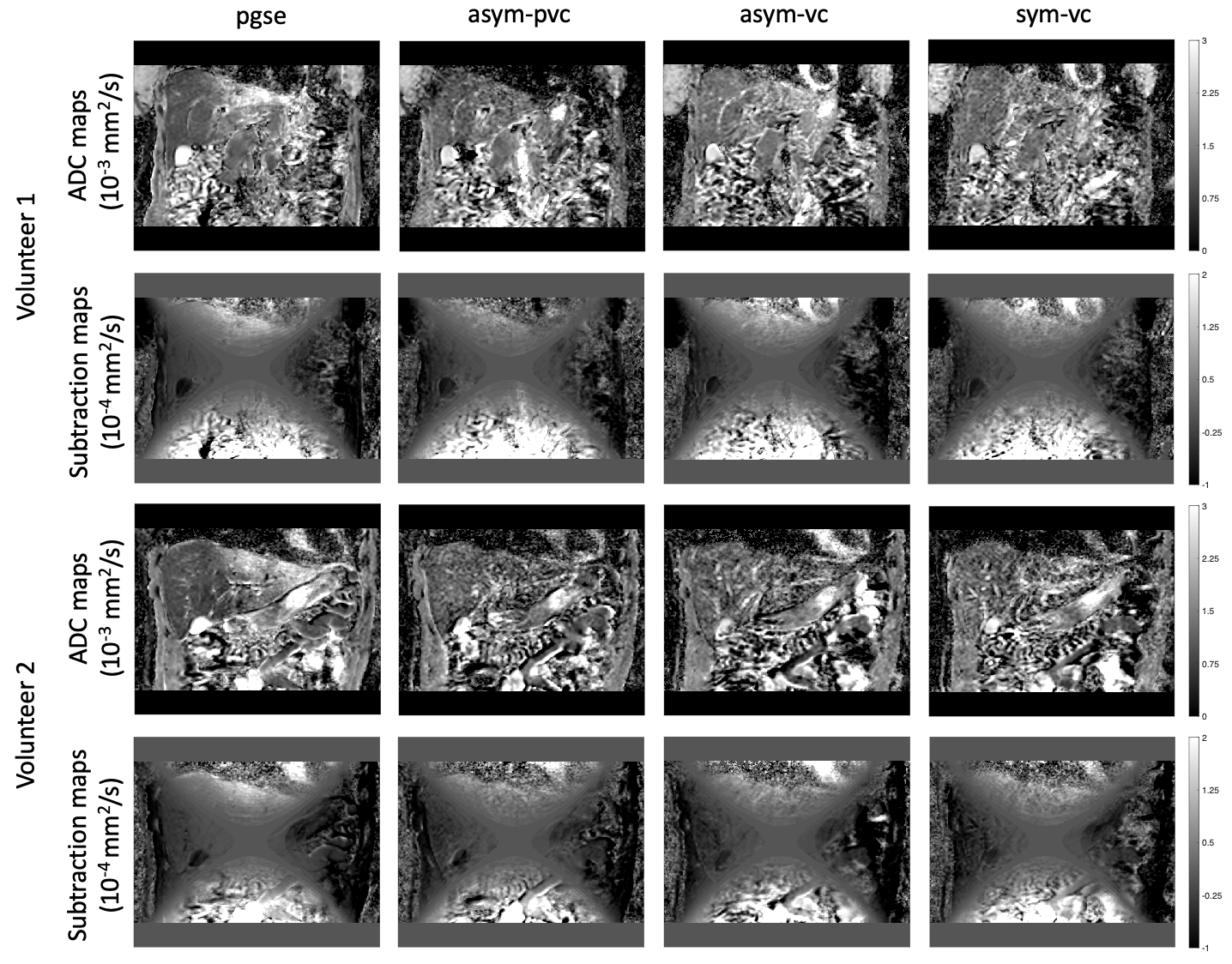


**Fig. S6** ADC maps and subtraction maps for all waveforms. The data from Volunteer 1 were acquired with diffusion encoding along the overplus directions, and the data from Volunteer 2 were acquired with diffusion encoding along the RO, PE and SS directions. The ADC maps for the motion compensated waveforms do not show as large of an ADC overestimation in the left liver lobe when compared with pgse. The subtraction maps therefore do not show as large of a difference between the gnl corrected and non-corrected ADC maps, apart from in regions where there are large vessels

**REFERENCES**

1. Aliotta E, Wu HH, Ennis DB (2017) Convex optimized diffusion encoding (CODE) gradient waveforms for minimum echo time and bulk motion compensated diffusion weighted MRI. Magn Reson Med 77:717-729.

2. Peña-Nogales Ó, Zhang Y, Wang X, de Luis-Garcia R, Aja-Fernández S, Holmes JH, Hernando D (2019) Optimized Diffusion-Weighting Gradient Waveform Design (ODGD) formulation for motion compensation and concomitant gradient nulling. Magn Reson Med 81:989-1003.

3. Baron CA, Lebel RM, Wilman AH, Beaulieu C (2012) The effect of concomitant gradient fields on diffusion tensor imaging. Magn Reson Med 68(4):1190-1201.

4. Bernstein MA, Zhou XJ, Polzin JA, King KF, Ganin A, Pelc NJ, Glover GH (1998) Concomitant gradient terms in phase contrast MR: analysis and correction. Magn Reson Med 39(2):300-308.
